# Effect of Formaldehyde on Human Middle Ear Epithelial Cells

**DOI:** 10.1155/2018/6387983

**Published:** 2018-03-26

**Authors:** Shin Hye Kim, Ji-Woong Choi, Myung-Whan Suh, Jun Ho Lee, Seung-Ha Oh, Jae-Jun Song, Moo Kyun Park

**Affiliations:** ^1^Department of Otorhinolaryngology-Head and Neck Surgery, Korea University Medical Center, Korea University College of Medicine, Seoul, Republic of Korea; ^2^Department of Otorhinolaryngology-Head and Neck Surgery, Haeundae Paik Hospital, Inje University College of Medicine, Busan, Republic of Korea; ^3^Wide River Institute of Immunology, Seoul National University College of Medicine, Gangwon, Republic of Korea; ^4^Department of Otorhinolaryngology-Head and Neck Surgery, Seoul National University Hospital, Seoul National University College of Medicine, Seoul, Republic of Korea; ^5^Sensory Organ Research Institute, Seoul National University Medical Research Center, Seoul, Republic of Korea

## Abstract

Formaldehyde (FA) is a familiar indoor air pollutant found in everything from cosmetics to clothing, but its impact on the middle ear is unknown. This study investigated whether FA causes cytotoxicity, inflammation, or induction of apoptosis in human middle ear epithelial cells (HMEECs). Cell viability was investigated using the trypan blue assay and a cell counting kit (CCK-8) in HMEECs treated with FA for 4 or 24 h. The expression of genes encoding the inflammatory cytokine tumor necrosis factor alpha (TNF-*α*) and mucin (MUC5AC) was analyzed using RT-PCR. Activation of the apoptosis pathway was determined by measuring mitochondrial membrane potential (MMP), cytochrome oxidase, caspase-9/Mch6/Apaf 3, and Caspase-Glo® 3/7 activities. The CCK-8 assay and trypan blue assay results showed a reduction in cell viability in FA-treated HMEECs. FA also increased the cellular expression of TNF-*α* and MUC5AC and reduced the activities of MMP and cytochrome oxidase. Caspase-9 activity increased in cells stimulated for 4 h, as well as caspase-3/7 activity in cells stimulated for 24 h. The decreased cell viability, the induction of inflammation and mucin gene expression, and the activation of the apoptosis pathway together indicate a link between environmental FA exposure and the development of otitis media.

## 1. Introduction

Otitis media (OM), or infection of the middle ear, is a common inflammatory disease among children. More than 80% of children will have one or more episodes of OM by the age of three [1]. OM leads to conductive hearing loss and may induce delays in the development of speech, language, balance, and learning [2]. Approximately 2.2 million children are diagnosed with OM each year, with the cost estimated at 3–5 billion USD in the USA alone [3, 4]. Therefore, identification and control of risk factors for OM will have a significant impact on healthcare costs.

Formaldehyde (FA) is a colorless, reactive, flammable gas with a strong odor. It is widely used in various household and industrial products and is both an indoor air pollutant and a byproduct of vehicle emissions and cigarette smoke [5]. Small amounts (<4 *μ*M) of FA are also produced endogenously by tumor cells [6]. FA causes adverse health effects in exposed humans and animals and since 2012 it has been classified as a Group 1 carcinogen (carcinogenic to humans) by the International Agency for Research on Cancer [7]. Acute exposure to FA can cause eye, nose, throat, and skin irritation, in addition to Alzheimer's disease-like changes in the brain [8]; long-term exposure has been associated with various cancers [7, 9].

FA also irritates the upper and lower respiratory tracts [10]. Several mechanisms by which FA might cause airway disease have been proposed. For example, FA may associate with protein molecules, such as albumin, to create new antigenic moieties [11]. This may in turn lead to the formation of specific IgE antibodies that bind to mast cells, followed by degranulation of such cells, and the release of mediators of inflammation [12, 13]. In addition, FA inhalation nonspecifically provokes airway mucosal inflammation [14, 15].

Middle ear disease may develop as an extension of upper airway disease, with the two anatomic sites linked through the Eustachian tube. This situation is more common in children because of their wider and more horizontal Eustachian tubes [16]. Consequently, a toxic substance in the upper airway can trigger middle ear disease, especially in children. The aim of this study was to investigate the effect of FA on cell viability and induction of inflammation in human middle ear epithelial cells (HMEECs). We also examined FA-induced activation of apoptosis.

## 2. Materials and Methods

### 2.1. Cell Culture

HMEECs were kindly provided by Dr. David J. Lim [17] and grown as described previously [18]. Briefly, Dulbecco's modified Eagle's medium (DMEM) and bronchial epithelial basal medium (BEBM) (1 : 1) mixed with supplements served as the growth medium. The cells were incubated at 37°C in a humidified atmosphere containing 95% air with 5% CO_2_. The growth medium was changed every third or fourth day. After 1 week, the cells were stimulated with different concentrations of FA (Sigma, St Louis, MO, USA) suspended in phosphate-buffered saline (PBS). As a control group, HMEECs were not treated with FA. This study was approved by the institutional review board of Seoul National University Hospital [H-1607-127-777].

### 2.2. Cell Viability

#### 2.2.1. Trypan Blue Assay

Trypan blue stains dead cells. Live cells possess intact membranes that exclude the dye, whereas dead cells do not. HMEECs were seeded in 96-well plates at 1 × 10^5^ cells/well and then incubated with 0, 0.5, 1, or 2 mM FA for 4 or 24 h. After three washes with PBS, 0.2% trypan blue solution (Gibco, Waltham, MA, USA) was added to each well. After incubation for 1 min at room temperature, the trypan blue solution was removed and 4% paraformaldehyde (PFA) was added to each well for 10 min. The cells were then washed three times with PBS. Viable cells were observed by microscopy with or without a green filter. The results were obtained from two repeated experiments using triplicate samples.

#### 2.2.2. CCK-8 Assay

We used a cell counting kit (CCK-8; Dojindo Laboratories, Kumamoto, Japan) to analyze cell viability and establish experimental conditions for the HMEEC assays. HMEECs were seeded into 96-well plates at 1.7 × 10^4^ cells/well. After 24 h, the cells were incubated with various concentrations of FA (0, 0.025, 0.05, 0.1, 0.2, 0.4, 0.6, 0.8, 1, 1.25, 1.5, or 2 mM) for 4 or 24 h. The cells were then washed three times with PBS, and the CCK-8 solution [10% (v/v)] was added to each well. The plates were incubated in the dark for 1 h at 37°C and then placed on a shaker at room temperature for 5 min. Optical densities were measured at 450 nm using a microplate reader (Cytation 5; BioTek, Winooski, VT, USA). The results are the means of three experiments, each performed in triplicate.

### 2.3. Expression of Genes Encoding the Inflammatory Cytokine (TNF-*α*) and Mucin (MUC5AC)

HMEECs were seeded in 96-well plates at 1.7 × 10^4^ cells/well. After 24 h, they were incubated with 0, 1.5, and 2 mM FA for 4 h and 0, 0.1, and 0.2 mM FA for 24 h. Total RNA was extracted from the HMEECs using an RNeasy® mini kit (Qiagen GmbH, Hilden, Germany). RNA (3 *μ*g) was reverse-transcribed at 55°C for 30 min and the resulting cDNAs were then amplified using a PCR kit (Qiagen, Valencia, CA, USA). The sequences of the oligonucleotide primers used in the PCRs were as follows:* TNF*, 5′-GGAGAAGGGTGACCGACTCA-3′;* MUC5AC*, 5′-CAGCACAACCCCTGTTTCAAA-3′; and* GAPDH*, 5′-ATGGCACCGTCAAGGCTGAG-3′. PCR amplification was performed under the following conditions: a holding stage at 95°C for 20 s followed by 45 cycles of 95°C for 1 s and 60°C for 20 s and then a melting stage at 95°C for 15 s and 60°C for 60 s. The results were obtained from three repeated experiments using triplicate samples. The data were analyzed using QuantStudio 6 Flex software (Applied Biosystems, Foster City, CA, USA).

### 2.4. Cell Apoptosis

#### 2.4.1. Measurement of Mitochondrial Membrane Potential (MMP)

HMEECs were incubated with 0, 1.5, or 2 mM FA for 4 h and 0, 0.1, or 0.2 *μ*M FA for 24 h and then mixed with 2 *μ*M 5′,6,6′-tetrachloro-1,1′,3,3′-tetraethylbenzimidazolylcarbocyanine iodide (JC-1) reagent. The stained cells were counted. To confirm a change in the JC-1 reaction due to a change in the MMP, a reference reaction with 50 *μ*M CCCP (carbonyl cyanide 3-chlorophenylhydrazone) was established. The stained HMEECs were analyzed using a flow cytometer with an excitation wavelength of 530/30 nm and 585/42 nm bandpass emission filters. Normal membrane potential was indicated by JC-1 red fluorescence and a loss of membrane potential by JC-1 green fluorescence. The loss of membrane potential (%) was calculated based on measurements from six samples.

#### 2.4.2. Cytochrome Oxidase Activity Colorimetric Assay

HMEECs were incubated with 0, 1.5, and 2 mM FA for 4 h and 0, 0.1, and 0.2 *μ*M FA for 24 h. Apoptosis was then analyzed using a cytochrome oxidase activity colorimetric assay kit (BioVision, Milpitas, CA, USA) after cell lysis. The change in absorbance at 550 nm via the oxidation of reduced cytochrome c, measured in six replicate samples, served as an indicator of cytochrome oxidase activity.

#### 2.4.3. Caspase-9/Mch6/Apaf-3 Colorimetric Protease Assay

HMEECs were incubated with 0, 1.5, and 2 mM FA for 4 h and 0, 0.1, and 0.2 *μ*M FA for 24 h. Apoptotic cells were quantified using an ADAM cell counter (ADAM MC; Digital Bio, Seoul, Korea), and the amount of cell protein was quantified in a bicinchoninic acid assay after cell lysis. Apoptosis markers were detected in real time using a caspase-9/Mch6/Apaf-3 colorimetric protease assay kit (Invitrogen, Carlsbad, CA, USA). Cells seeded in 96-well plates were scanned and their optical density (OD) at 405 nm was measured using a microreader (Cytation 3; BioTek, Winooski, VT, USA). The relative absorbance of six FA-treated versus untreated samples was determined.

#### 2.4.4. Caspase-Glo 3/7 Assay

HMEECs were incubated with 0, 1.5, and 2 mM FA for 4 h and 0, 0.1, and 0.2 *μ*M FA for 24 h. Apoptosis activity was analyzed using a Caspase-Glo 3/7 assay kit (Promega, Madison, WI, USA). Following caspase cleavage, a substrate that reacts with luciferase (aminoluciferin) is released and the light produced in the reaction is then measured. In this study, the luminescence of six FA-treated and untreated samples was measured at 0.5, 1.0, 1.5, 2.0, 2.5, and 3.0 h using a microplate reader.

### 2.5. Statistical Analysis

Statistical significance was determined with the aid of the one-tailed paired-sample *t-test*. A *P* value < 0.05 for the null hypothesis was considered to indicate a statistically significant difference. SPSS software (ver. 11.0; SPSS Inc., Chicago, IL, USA) was used for the statistical analysis.

## 3. Results

### 3.1. FA Reduces the Viability of HMEECs

**Figure 1:**
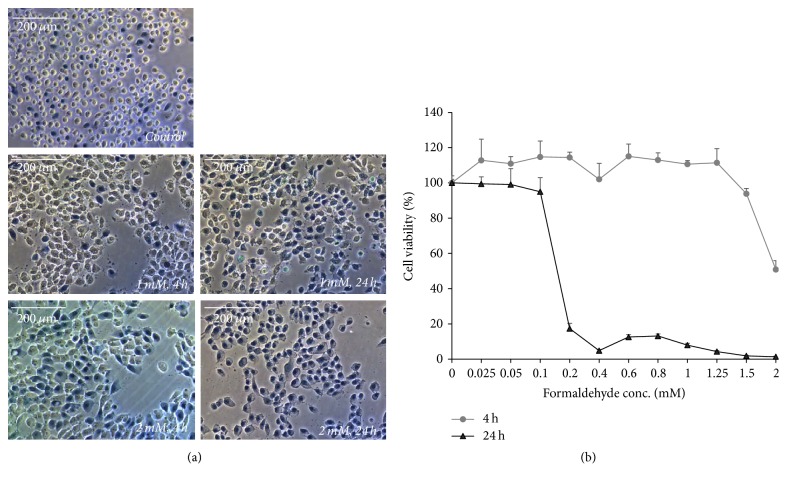
*Viability of human middle ear epithelial cells (HMEECs) following formaldehyde (FA) exposure*. (a) Nonviable cells take up trypan blue and their numbers increase to greater extents in cultures exposed to FA for 24 h versus 4 h and to 2 mM versus 1 mM FA (×200, trypan blue assay without green filter). (b) The cell counting kit- (CCK-) 8 assay revealed that cell viability decreased after cells were exposed to 1.5 mM FA for 4 h and 0.2 mM FA for 24 h. Error bars represent standard deviation (SD) of the mean.

In the trypan blue assay, dead cells were stained with trypan blue. The number of viable cells decreased with increasing FA concentrations and longer FA exposure time compared to untreated control cells (Figure 1(a)). Moreover, there were fewer viable cells in cultures subjected to 2 mM FA for 24 h than in cultures incubated with 1 mM FA for 4 h.

The CCK-8 assay of HMEECs treated with 0.025–1.25 mM FA for 4 h showed that cell viability was maintained at almost 100% but decreased to 50.9% in cells exposed to 2 mM FA (Figure 1(b)). In HMEECs treated with 0.2 mM FA for 24 h, viability rapidly decreased to <20%. Thus, in the following experiments, these concentrations were considered appropriate when performing the 4 and 24 h exposure experiments. The 50% lethal concentration (LC_50_) of FA was 2.038 mM over 4 h and 0.154 mM over 24 h.

### 3.2. FA Increases Inflammatory Cytokine and Mucin Gene Expression in HMEECs

**Figure 2:**
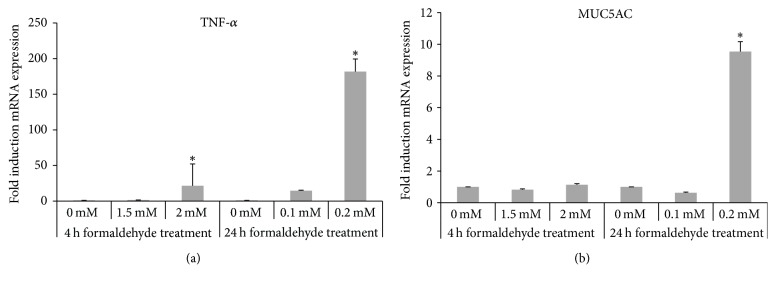
*Upregulation of the inflammatory cytokine tumor necrosis factor alpha (TNF-α) and mucin gene (MUC5AC) expression in HMEECs exposed to FA*. (a) The expression of mRNA encoding TNF-*α* was upregulated in cells treated with 2 mM FA for 4 h and 0.2 mM FA for 24 h. (b) The expression of mRNA encoding MUC5AC was upregulated in cells treated with 0.2 mM FA for 24 h. Error bars represent SD of the mean. Data represent the means ± SD from three repeated experiments with triplicate samples. ^*∗*^*P* < 0.05 compared with the control values.

Significant increases in* TNF-α* gene expression in HMEECs exposed to 2 mM FA for 4 h and to 0.2 mM FA for 24 h were determined (Figure 2(a)). The expression of the* MUC5AC* gene in HMEECs increased significantly as well as when exposed to 0.2 mM FA for 24 h (Figure 2(b)).

### 3.3. FA Increases the Loss of MMP in HMEECs

**Figure 3:**
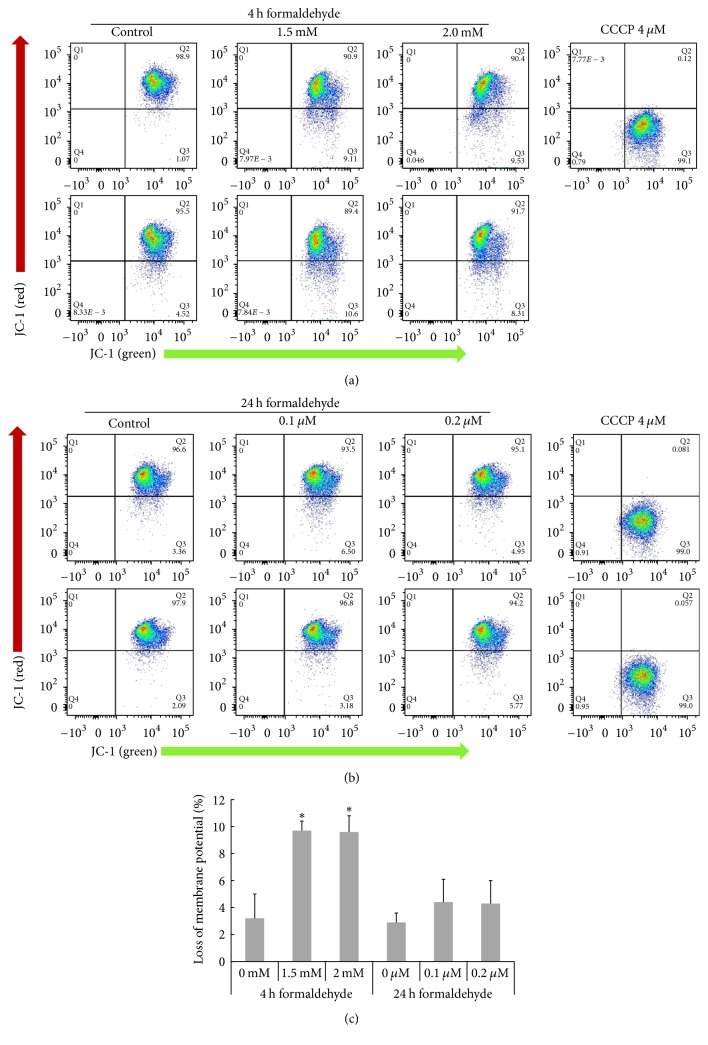
*Loss of mitochondrial membrane potential (MMP) in HMEECs following FA exposure*. (a, b) Histogram of a MitoProbe^™^ JC-1 assay shows the decreased red/green fluorescence intensity ratios following cell exposure to FA for 4 h or 24 h. (c) HMEECs exhibited reductions in MMP levels after 4 or 24 h of FA treatment. After 4 h of FA treatment, the reduction in MMP level was statistically significant. Error bars represent SD of the mean. The data represent the means ± SD of three repeated experiments with six samples. ^*∗*^*P* < 0.05 compared with control.

When exploring MMP- and apoptosis-related activity, cells were exposed to 0.1 and 0.2 *μ*M FA (rather than 0.1 and 0.2 mM FA) for 24 h, because exposure to 0.1 or 0.2 mM FA for 24 h induced cell fixation. JC-1 red (normal membrane potential) and JC-1 green (loss membrane potential) fluorescence were analyzed using flow cytometry (Figures 3(a) and 3(b)). The loss of MMP was significant in cells stimulated with 1.5 and 2 mM FA for 4 h (9.9% and 8.9%, resp.); smaller losses occurred in response to 0.1 and 0.2 *μ*M FA for 24 h (4.9% and 5.4%, resp.) (Figure 3(c)).

### 3.4. FA Increases Apoptosis in HMEECs

**Figure 4:**
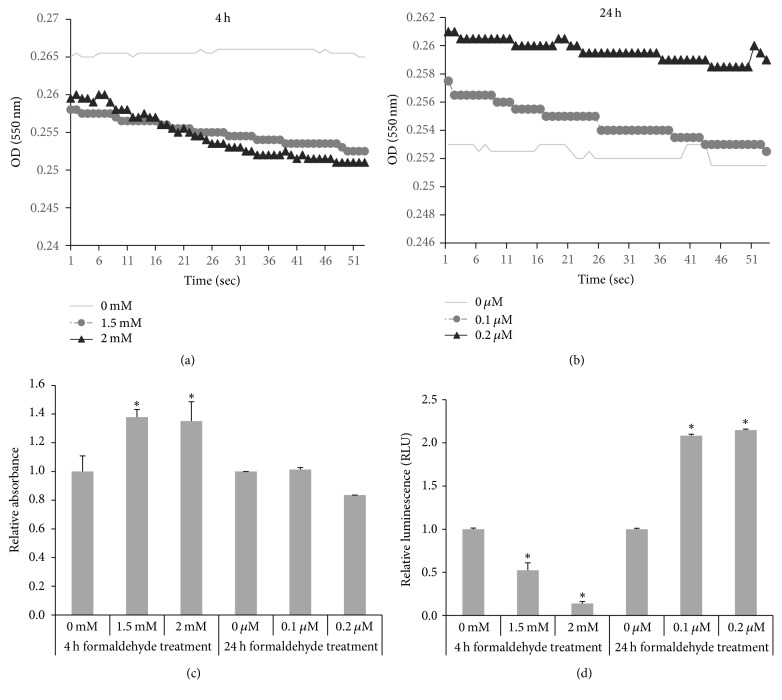
*Apoptosis pathway activity in HMEECs exposed to FA*. (a, b) Cytochrome oxidase activity decreased after 4 h of FA exposure and increased after 24 h of FA exposure in a dose-dependent manner. (c) Caspase-9/Mch6/Apaf-3 activity, measured as the relative absorbance, increased in cells stimulated for 4 h with 1.5 and 2 mM FA. (d) Caspase-Glo 3/7 activity, measured as relative luminescence, decreased after 4 h of FA exposure but increased after 24 h of exposure to FA. Error bars represent the SDs of the mean. The data represent the averages (a, b) or means ± SD (c, d) of three experiments, each with six replicates. ^*∗*^*P* < 0.05 compared with control.

Cytochrome oxidase activity was measured in HMEECs exposed to 0, 1.5, and 2 mM FA for 4 h and to 0, 0.1, and 0.2 *μ*M FA for 24 h (Figures 4(a) and 4(b)). After the 4 h of FA treatment, the luminescence levels were lower than those of controls at the higher FA concentrations. After 24 h of FA treatment, the luminescence was greater at the higher FA concentration compared to that of the control. To determine whether FA exposure activates the caspase cascade in HMEECs, the functional activities of the apoptosis initiator caspase-9 and the apoptosis effector caspase-3/7 were measured in HMEECs exposed to 0, 1.5, or 2 mM FA for 4 h or to 0, 0.1, or 0.2 *μ*M FA for 24 h. In the caspase-9 assay, the relative absorbance after 4 h of exposure to FA was greater at the higher FA concentration (Figure 4(c)). In the caspase-3/7 assay, the extent of luminescence after 24 h of exposure to FA was greater at the higher FA concentration (Figure 4(d)). Although the FA concentration during the 24 h treatment was 1/1,000-fold of that during the 4 h FA treatment, the luminescence levels indicated that caspase-3/7 activity levels were higher.

## 4. Discussion

In this study, we demonstrated that FA affects the viability of HMEECs even at low-dose FA (0.2 mM) during a long exposure (24 h). Under those conditions, cytotoxicity was greater than after a short exposure (4 h) to high-dose FA (2 mM). The endogenous concentration of FA in human blood is 80–100 *μ*M [19]. Thus, a prolonged exposure to a small additional amount of FA could lead to cytotoxicity in humans. FA affects cell morphology, causing cell shrinkage and decreasing the colony size of human NK cells within 1 h [20]. FA at concentrations of 0.1–0.2 mM also induces nuclear fragmentation and chromatin condensation, as hallmarks of apoptosis, in neuroblastoma cells [21]. Endogenous FA reacts with proteins to form immunogenic and atherogenic adducts (epitopes) [13]. FA cytotoxicity is explained in part by the fact that FA forms adducts with DNA and proteins [9, 22].

Our study further demonstrated that FA induces inflammatory cytokine* (TNF-α)* and mucin* (MUC5AC)* gene expression in HMEECs. Inflammatory cytokines including TNF-*α*, cyclooxygenase-2 (COX-2), nuclear factor- (NF-) *κ*B, interleukin- (IL-) 1, IL-6, and IL-8 play important roles in the initiation of mucosal changes, stimulation of mucin secretion, and immune regulation seen in OM [23–25]. We previously reported increased inflammatory cytokine and mucin gene expression in HMEECs exposed to environmental pollutants [18, 25, 26]. Some materials toxic to lung epithelium are also toxic to the middle ear mucosa because of the similar composition of these two tissues. For example, acrolein induces an inflammatory response and increases mucin gene expression in both human lung epithelial cells and HMEECs [26]. In murine alveolar macrophages immediately after FA exposure, several cytokines (IL-4, IL-10, IFN-*γ*, and TNF-*α*) became detectable by ELISA of cell supernatants [27]. FA aggravates asthma, at least in part by increasing the T helper-2 dominant response, which is related to levels of the cytokine mediators of asthma (IL-4, IL-5, IL-9, and IL-13) [12]. In addition, FA interferes with thiols, leading to accelerated reduction of the endogenous bronchodilator S-nitrosoglutathione [14]. Also, the Rho kinase-dependent Ca^2+^ sensitization pathway plays a role in the airway hyperresponsiveness induced by FA [28].

In this study, MMP decreased and apoptosis-related activity increased in FA-treated HMEECs. That the cytotoxicity of FA is related to the induction of apoptosis has previously been demonstrated in tumor cells, endothelial cell cultures, neural cells, and NK cells [20–22]. In a previous experiment, we showed that FA increases apoptosis (data not shown), but very high doses of FA result in necrotic cell death, especially coagulation necrosis, but also lytic cell death [22]. FA-mediated apoptosis is characterized by a marked decrease in MMP, inhibition of mitochondrial respiration, and formation of reactive oxygen species [29]. A decrease in MMP occurs in response to many apoptotic stimuli and is linked to the release of cytochrome c during apoptosis [30]. In this study, MMP loss was higher in cells treated with 1.5 or 2 mM FA for 4 h than in those treated with 0.1 or 0.2 *μ*M FA for 24 h.

In the HMEECs examined in our study, low-dose (0.1–0.2 *μ*M) FA increased cytochrome oxidase and caspase-7/3 activity in cells exposed for 24 h. However, after 4 h of exposure to 1.5 and 2 mM FA, reduced levels of both cytochrome oxidase and caspase-7/3 activity were measured. Cytochrome c interacts with apoptotic protease activating factor 1 (APAF1), which activates caspase-9. Active caspase-9, in turn, activates caspase-3 and caspase-7, eventually leading to apoptosis [31]. Accordingly, low cytochrome oxidase activity in cells exposed to FA for 4 h may diminish caspase-7/3 activity, although the relationship between cytochrome oxidase and the loss of MMP is not understood. A previous study showed that FA inhibits NADH dehydrogenase (complex I) and cytochrome c oxidase (complex IV) [21]. FA is commonly used for histological fixation of tissues and cultured cells; the effects of FA increase over time [32]. We found that some cells were fixed after exposure to 0.1–0.2 mM FA for 24 h. To minimize cell fixation caused by high concentrations of FA and to accurately measure the extent of apoptosis, we exposed cells to 0.1–0.2 *μ*M FA (rather than 0.1–0.2 mM FA) in the 24 h experiments, evaluating MMP status and apoptosis-related activity.

FA is highly water-soluble, existing in tissues as a reversibly hydrated form (methanediol) [33]. Most FA exposures occur by inhalation or skin/eye contact [33]. For the same level of FA, children may experience a larger exposure than adults because of the greater surface area of the pediatric lung [11]. Furthermore, because higher levels of FA occur nearer to the ground (lower specific gravity than air), short stature children may be exposed to higher levels than adults in the same location [11]. FA is highly reactive at the site of entry and reacts readily with macromolecules, including DNA, to form DNA-protein and DNA-DNA cross-links [34]. Inhaled FA is absorbed primarily in the upper airways because of its high water solubility, metabolism, and reactivity [35]. Diffusion through the mucus layer is the dominant transport mechanism for FA. Once in the mucus layer, FA undergoes a reversible reaction with water to form methanediol [33]. A proportion of inhaled FA passes through the mucus layer to reach the epithelium, wherein FA undergoes enzymatic transformation and removal in nasal tissue, and engages in nonenzymatic reactions with glutathione and macromolecules, including proteins and DNA [33]. The Rho kinase-dependent Ca^2+^ sensitization pathway plays a role in airway hyperresponsiveness to FA [28].

Environmental exposure to FA is a frequent occurrence but treatment is limited to supportive care, including decontamination (flushing of skin and eyes with water, gastric lavage, and administration of activated charcoal), administration of supplemental oxygen, intravenous sodium bicarbonate and/or isotonic fluid, and hemodialysis [36]. Therefore, greater efforts should be made to prevent exposure to FA, based on an awareness of its toxicity.

## 5. Conclusions

Our study proposes a link between environmental FA exposure and OM development. FA increases* TNF-α* and* MUC5AC* mRNA expression in HMEECs. Both mediators are increased in OM. FA decreases cell viability, increases the activity of the apoptosis pathway, causes a significant loss of MMP, and increases cytochrome oxidase, caspase-9, and caspase-3/7 activities.

## Additional Points

The English language in this document has been checked by at least two professional editors, both native speakers of English. For a certificate, please see http://www.textcheck.com/certificate/6VvQB2.
